# Targeted metabolomic analysis of serum amino acids in the adult Fontan patient with a dominant left ventricle

**DOI:** 10.1038/s41598-020-65852-x

**Published:** 2020-06-02

**Authors:** Miriam Michel, Karl-Otto Dubowy, Andreas Entenmann, Daniela Karall, Mark Gordian Adam, Manuela Zlamy, Irena Odri Komazec, Ralf Geiger, Christian Niederwanger, Christina Salvador, Udo Müller, Kai Thorsten Laser, Sabine Scholl-Bürgi

**Affiliations:** 1 Department of Pediatrics III, Division of Pediatric Cardiology, Medical University of Innsbruck, Anichstraße 35, 6020 Innsbruck, Austria; 2 Center of Pediatric Cardiology and Congenital Heart Disease, Heart and Diabetes Center North Rhine-Westphalia, Ruhr-University of Bochum, Georgstraße 11, 32545 Bad Oeynhausen, Germany; 3 Department of Pediatrics I, Division of Gastroenterology and Hepatology, Medical University of Innsbruck, Anichstraße 35, 6020 Innsbruck, Austria; 4 Department of Pediatrics I, Division of Inherited Metabolic Disorders, Medical University of Innsbruck, Anichstraße 35, 6020 Innsbruck, Austria; 5 Biocrates Life Sciences AG, Eduard-Bodem-Gasse 8, 6020 Innsbruck, Austria

**Keywords:** Congenital heart defects, Medical research

## Abstract

Growing interest lies in the assessment of the metabolic status of patients with a univentricular circulation after Fontan operation, especially in changes of amino acid metabolism. Using targeted metabolomic examinations, we investigated amino acid metabolism in a homogeneous adult Fontan-patient group with a dominant left ventricle, seeking biomarker patterns that might permit better understanding of Fontan pathophysiology and early detection of subtle ventricular or circulatory dysfunction. We compared serum amino acid levels (42 analytes; AbsoluteIDQ p180 kit, Biocrates Life Sciences, Innsbruck, Austria) in 20 adult Fontan patients with a dominant left ventricle and those in age- and sex-matched biventricular controls. Serum concentrations of asymmetric dimethylarginine, methionine sulfoxide, glutamic acid, and trans-4-hydroxyproline and the methionine sulfoxide/methionine ratio (Met-SO/Met) were significantly higher and serum concentrations of asparagine, histidine, taurine, and threonine were significantly lower in patients than in controls. Met-SO/Met values exhibited a significant negative correlation with oxygen uptake during exercise. The alterations in amino acid metabolome that we found in Fontan patients suggest links between Fontan pathophysiology, altered cell energy metabolism, oxidative stress, and endothelial dysfunction like those found in biventricular patients with congestive heart failure. Studies of extended amino acid metabolism may allow better understanding of Fontan pathophysiology that will permit early detection of subtle ventricular or circulatory dysfunction in Fontan patients.

## Introduction

Ventricular dysfunction and circulatory failure with progressing end-organ impairment like renal or liver dysfunction are an important cause of morbidity and mortality in adults with complex congenital heart disease (CHD), especially in patients with single-ventricle types of CHD and Fontan circulation^1,2^. Besides limited cardiac output, alterations that mark Fontan hemodynamics are passive flow to the lungs, chronically elevated venous pressures, and congestion. Unfortunately, the clinical use of traditional markers such as N-terminal prohormone of brain natriuretic peptide (NT-proBNP) levels for non-invasive diagnostics and monitoring in such patients is limited^3,4^. Thus, for early detection of cardiac and circulatory derangement and for evaluation and tailoring of treatment options, regular functional assessment of these patients is crucial, with complete clinical examination, electrocardiogram, imaging studies, determination of values for traditional laboratory markers, or exercise capacity testing. In adult biventricular patients, novel candidate biomarkers, in addition to natriuretic peptides and troponins, for congestive heart failure and vascular perturbations have been identified via metabolomics, the study of small organic molecules, their synthesis, and their breakdown^5,6^. Interest has grown recently in the metabolic status of Fontan patients: besides reported abnormalities in Fontan patients’ glucose metabolism^7^, to date their handling of lipids has been best studied, with important changes shown, especially in the cholesterol, lipoprotein, and phospholipid pathways, hinting at chronic low-level inflammation and at altered cell signalling and energy metabolism as are found in biventricular patients with congestive heart failure^8,9^. In the biventricular patient with heart failure, alterations occur especially in the handling of amino acids important in both myocardium protein turnover and energy metabolism^10^. Additionally, the Fontan-specific characteristic of chronically elevated venous pressure favors development of a protein losing enteropathy together with end-organ, especially liver, dysfunction^2^. Thus, study of the metabolism of amino acids also should be a promising field in Fontan patients. With the help of targeted metabolomic examinations we hence elected to investigate amino acid metabolism in a homogeneous adult Fontan-patient group with a dominant left ventricle, seeking biomarker patterns that might permit a better understanding of Fontan pathophysiology or early detection of ventricular or circulatory dysfunction.

## Results

After applying all inclusion and exclusion criteria, 20 adult Fontan patients with a systemic left ventricle were selected for the study (Supplemental Fig. 1). The results of “traditional” examinations (patient and control clinical assessment, exercise capacity testing, routine laboratory analyses) are set out in our recent work on lipid metabolism^9^. Table 1 lists clinical and exercise capacity testing parameters, showing as major features that in Fontan patients, minimum (at exercise) and maximum (at rest) pulse-oximeter oxygen saturation as well as $\dot{{\rm{V}}}$O_2_ at the anaerobic threshold and at maximum were significantly lower than in controls.

**Table 1:** Participants’ clinical characteristics^9^.

	Fontan patients	Controls	P-value
Total [n]	20	20	
Female sex [n]	7	7	
Age [years]	23.1 ± 5.1	24.7 ± 6.6	0.28
After TCPC [years]	18.8 ± 5.2		
Bodyweight [kg]	69.8 ± 13.2	73.3 ± 11.7	0.17
Height [cm]	171.3 ± 7.4	174.5 ± 8.7	0.04
Body mass index [kg/m²]	23.8 ± 4.1	22.5 ± 3.3	0.05
SpO_2_ at rest [%]	93 ± 3	99 ± 1	<0.00001
SpO_2_ at exercise [%]	90 ± 3	98 ± 1	<0.00001
$\dot{{\rm{V}}}$O_2_ at rest [mL/kg/min]	5.6 ± 1.7	5.8 ± 1.1	0.03
$\dot{{\rm{V}}}$O_2_AT [mL/kg/min]	24.5 ± 4.9	30.1 ± 3.6	<0.00001
Peak $\dot{{\rm{V}}}$O_2_ [mL/kg/min]	28.8 ± 10.1	45.7 ± 6.4	<0.00001
Double inlet left ventricle [n]	10		
TA + PS/PA [n]	9		
TA + PS + VSD [n]	1		
Extracardiac Fontan [n]	16		
Open fenestration (at study) [n]	3		
LPA dilation/stent [n]	4		
Tunnel dilation/stent [n]	6		
Closure of fenestration [n]	1		
Closure of vv collateral [n]	3		
Electrophysiologic examination [n]	2		

Values are given as mean ± standard deviation. AT, anaerobic threshold; LPA, left pulmonary artery; n, number; PA, pulmonary atresia; PS, pulmonary stenosis; SpO_2_, pulsoxymetric oxygen saturation; TA, tricuspid atresia; TCPC, total cavopulmonary connection; $\dot{{\rm{V}}}$O_2_, oxygen uptake; VSD, ventricular septal defect; vv, veno-venous.

### Routine analytes

Hematocrit, hemoglobin concentrations (“hemoglobin”), gamma glutamyl transferase and alanine aminotransferase activities, total bilirubin and creatinine concentrations (“total bilirubin” and “creatinine”), and international normalized ratio (INR) values and triglyceride and high density lipoprotein-cholesterol (HDL-C) concentrations (“triglycerides” and “HDL-C”) differed significantly between Fontan patients and controls (Table 2).

**Table 2:** Values of routine analytes and of amino acids or biogenic amines and their derivatives.

	Fontan patients	Controls	P-value
Hematocrit [%]	47.8 ± 5.6	39.3 ± 4.2	<0.00001↑
Hemoglobin [g/dL]	16.4 ± 2.1	12.7 ± 1.4	<0.00001↑
Total cholesterol [mg/dL]	145.3 ± 26.5	149 ± 34.2	0.77
HDL-C [mg/dL]	42.5 ± 15.9	51.3 ± 12.3	0.03↓
Non-HDL-C [mg/dL]	85.2 ± 24.8	73.1 ± 20.8	0.2
Triglycerides [mg/dL]	128.6 ± 86.5	47.3 ± 22.8	0.003↑
Total protein [g/dL]	7.2 ± 0.5	7.0 ± 0.7	0.31
Albumin [mg/dL]	4145 ± 492	4215 ± 208	0.64
Creatinine [mg/dL]	0.8 ± 0.12	0.53 ± 0.18	<0.00001↑
Total bilirubin [mg/dL]	1.22 ± 0.67	0.3 ± 0.29	<0.00001↑
AST [U/L]	35.3 ± 7.7	31.6 ± 8.4	0.12
ALT [U/L]	39.4 ± 11.4	31.9 ± 10.1	0.04↑
gGT [U/L]	86.5 ± 43.6	35.1 ± 19.4	0.00002↑
INR	2.1 ± 0.76	1.02 ± 0.04	<0.00001↑
NT-proBNP [pg/mL]	52.4 ± 69.2	39.3 ± 30.4	0.88
CRP [mg/dL]	0.18 ± 0.2	0.16 ± 0.14	0.47

Values are given as mean ± standard deviation. ALT, alanine aminotransferase; AST, aspartate aminotransferase; CRP, C-reactive protein; dL, decilitre; g, gram; gGT, gamma glutamyl transferase; HDL-C, high density lipoprotein-cholesterol; INR, international normalized ratio; NT-proBNP, N-terminal prohormone of brain natriuretic peptide; U, unit; ↑, statistically significant higher serum concentration in Fontan patients than in controls; ↓, statistically significant lower serum concentration in Fontan patients than in controls.

### Metabolomic examination of serum amino acids

**Figure 1:**
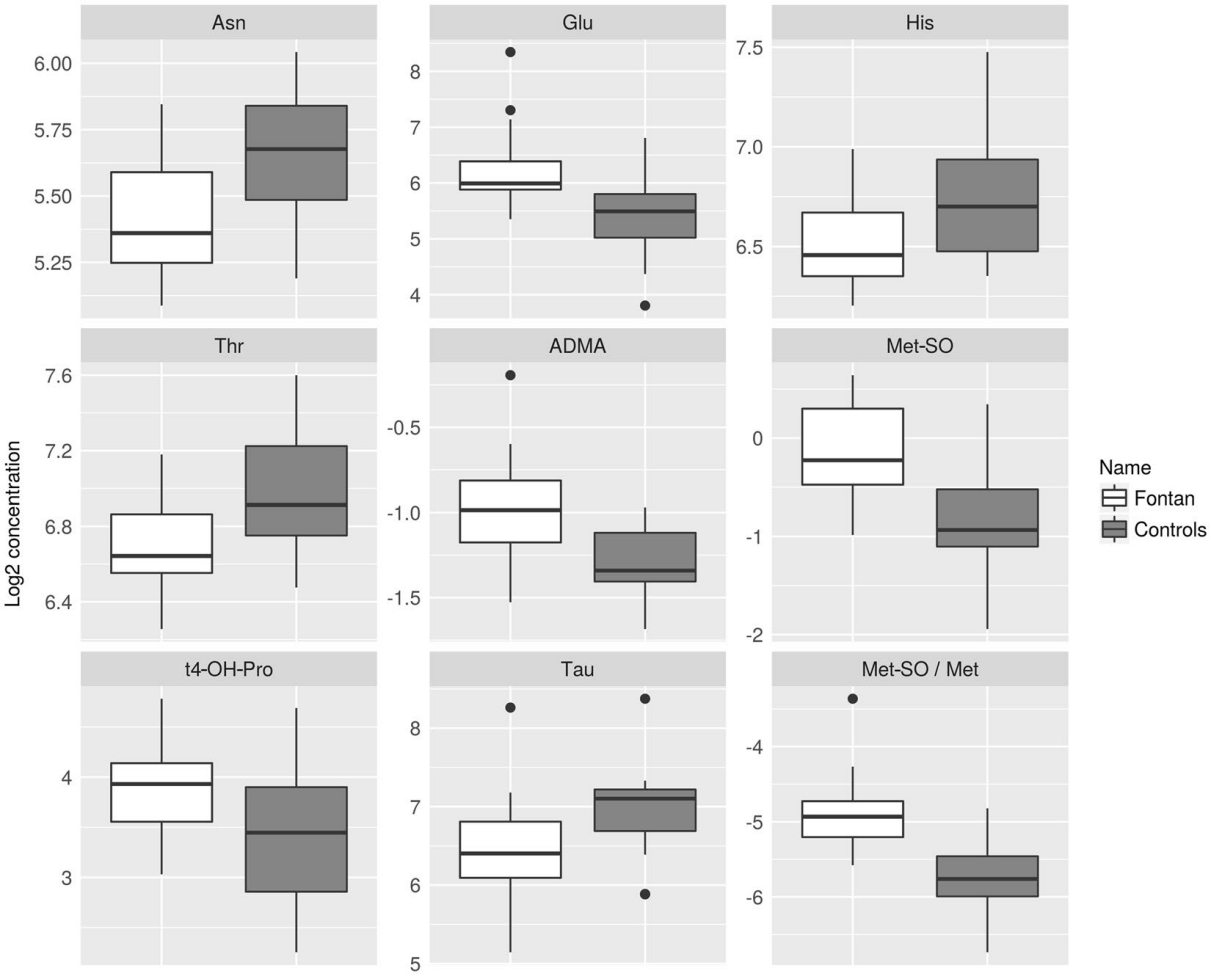
Box-and-whisker plots of serum concentrations of amino acids or biogenic amines and their ratios that differed significantly between Fontan patients (grey boxes) and controls (white boxes). The boxes show the 1^st^ (Q1) and 3^rd^ quartile (Q3), the whiskers the minimum and the maximum. Black circles represent outlying values as identified by the interquartile range (IQR) rule (values smaller than (Q1-1.5*IQR) or values larger than (Q3 + 1.5*IQR)). ADMA, asymmetric dimethylarginine; Asn, asparagine; Glu, glutamic acid; His, histidine; Met, methionine; Met-SO, methionine sulfoxide; Met-SO/Met, methionine sulfoxide/methionine ratio; t4-OH-Pro, trans-4-hydroxyproline; Tau, taurine; Thr, threonine.

Serum concentrations of 29 amino acids or biogenic amines and their derivatives were determined, with selected sums and ratios (Table 3, Fig. 1). In Fontan patients serum concentrations of methionine (Met) sulfoxide (Met-SO), asymmetric dimethyl arginine (ADMA), glutamic acid, and trans-4-hydroxyproline and the ratio of Met-SO to Met (Met-SO/Met) were significantly higher, and serum concentrations of asparagine, histidine, taurine, and threonine were significantly lower than in controls.

**Table 3:** Values of amino acids or biogenic amines and their derivatives, and of selected sums and ratios.

Metabolite	HMBD ID	Patients	Controls	p-value	q-value	mean FC
Ac-Orn	HMDB0003357	NA	NA	NA	NA	NA
ADMA	HMDB01539	0.51 ± 0.12	0.41 ± 0.05	0.0004	0.002↑	1.26
Alanine	HMDB00161 HMDB01310	401.4 ± 84.2	410.5 ± 123.0	0.99	0.99	−1.02
alpha-AAA	HMDB00510	1.12 ± 0.61	0.77 ± 0.44	0.03	0.06	1.44
Arginine	HMDB00517 HMDB03416	91.7 ± 26.8	108.2 ± 21.8	0.03	0.06	−1.18
Asparagine	HMDB00168 HMDB003378	43.7 ± 7.7	50.7 ± 8.0	0.007	0.016↓	−1.16
Aspartic acid	HMDB00191 HMDB06483	18.0 ± 10.4	17.2 ± 6.9	0.93	0.96	1.05
c4-OH-Pro	HMDB0240251	NA	NA	NA	NA	NA
Carnosine	HMDB00033	NA	NA	NA	NA	NA
Citrulline	HMDB00904	29.4 ± 6.3	29.2 ± 6.6	0.91	0.95	1.01
DOPA	HMDB00181 HMDB00609	NA	NA	NA	NA	NA
Dopamine	HMDB00073	NA	NA	NA	NA	NA
Glutamine	HMDB00641 HMDB03423	717.4 ± 134.1	749.0 ± 111.7	0.38	0.47	−1.04
Glutamic acid	HMDB00148 HMDB03339	85.9 ± 63.4	47.0 ± 21.8	0.0006	0.002↑	1.83
Glycine	HMDB00123	321.6 ± 74.1	345.3 ± 91.7	0.41	0.5	−1.07
Histidine	HMDB00177	93.1 ± 15	110.4 ± 24.4	0.008	0.02↓	−1.19
Histamine	HMDB00870	NA	NA	NA	NA	NA
Isoleucine	HMDB00172 HMDB0000557	106.6 ± 31.6	100.9 ± 39.5	0.44	0.53	1.06
Kynurenine	HMDB00684	NA	NA	NA	NA	NA
Leucine	HMDB00687 HMDB0013773	222.9 ± 78.6	232.8 ± 106.6	0.99	0.99	−1.04
Lysine	HMDB00182 HMDB03405	168.1 ± 23.5	165.8 ± 32.4	0.69	0.79	1.01
Methionine	HMDB00696	27.9 ± 8.3	29.4 ± 7.8	0.47	0.55	−1.06
Met-SO	HMDB02005	0.94 ± 0.32	0.58 ± 0.23	0.00008	0.0005↑	1.64
Nitro-Tyr	HMDB01904	NA	NA	NA	NA	NA
Ornithine	HMDB00214 HMDB03374	139.4 ± 82.3	111.6 ± 40.9	0.22	0.29	1.25
PEA	HMDB0012275	NA	NA	NA	NA	NA
Phenylalanine	HMDB00159	77.7 ± 16.9	77.0 ± 12.9	0.99	0.99	1.01
Proline	HMDB00162 HMDB03411	259.4 ± 63.7	265.7 ± 61.6	0.73	0.81	−1.02
Putrescine	HMDB01414	NA	NA	NA	NA	NA
Sarcosine	HMDB00271	1.57 ± 0.94	1.62 ± 0.79	0.73	0.81	−1.03
SDMA	HMDB03334	0.46 ± 0.06	0.42 ± 0.08	0.1	0.15	1.08
Serine	HMDB00187 HMDB03406	158.4 ± 33.3	146.5 ± 23.7	0.21	0.29	1.08
Serotonin	HMDB00259	0.56 ± 0.33	0.7 ± 0.21	0.03	0.06	−1.26
Spermidine	HMDB01257	NA	NA	NA	NA	NA
Spermine	HMDB01256	NA	NA	NA	NA	NA
t4-OH-Pro	HMDB00725 HMDB0006055	15.8 ± 5.06	11.7 ± 5.2	0.007	0.02↑	1.35
Taurine	HMDB00251	100.4 ± 57.1	132.6 ± 56.54	0.02	0.049↓	−1.32
Threonine	HMDB04041 HMDB00167	105.6 ± 21.0	127.3 ± 28.4	0.008	0.02↓	−1.21
Tryptophan	HMDB00929 HMDB0013609	90.5 ± 20.2	89.5 ± 23.3	0.84	0.89	1.01
Tyrosine	HMDB00158	87.0 ± 23.0	83.6 ± 19.4	0.69	0.79	1.04
Valine	HMDB00883	298.9 ± 72.5	290.7 ± 76.9	0.7	0.79	1.03
BCAA		628 ± 174	624 ± 217	0.8	0.87	1.01
AAA		255 ± 53	250 ± 47	0.8	0.87	1.02
Total AA		3544 ± 466	3588 ± 682	0.93	0.96	−1.01
Essential AA		1098 ± 223	1113 ± 293	0.97	0.99	−1.01
Fischer ratio		2.47 ± 0.52	2.45 ± 0.5	0.9	0.95	1.01
Met-SO/Met		0.04 ± 0.016	0.02 ± 0.007	<0.00001	0.0001↑	1.78

Metabolite concentrations for all analytes with selected sums and ratios, grouped by Fontan patients vs. controls. Values are given as mean ± standard deviation, unit of values of data [µmol/l]. FC, fold change (Fontan patients vs. controls); HMBD ID, Human Metabolome Database identification; AA, amino acids; AAA, aromatic amino acids; Ac-Orn, acetylornithine; ADMA, asymmetric dimethylarginine; alpha-AAA, alpha aminoadipic acid; BCAA, branched-chain amino acids; c4-OH-Pro, cis-4-hydroxyproline; DOPA, dihydroxyphenylalanine; Met-SO, methionine sulfoxide; Nitro-Tyr, nitrotyrosine; PEA, phenylethylamine; SDMA, symmetric dimethylarginine; t4-OH-Pro, trans-4-hydroxyproline; ↑, statistically significant higher serum concentration in Fontan patients than in controls; ↓, statistically significant lower serum concentration in Fontan patients than in controls. ‘Fischer ratio’ is the ratio of BCAA/AAA.

### Correlation of routine biochemical and clinical findings with metabolomic parameters

Among routine analytes, the variables hemoglobin, albumin, and triglycerides displayed significantly positive correlations with glutamic acid. Creatinine and triglycerides displayed significantly positive correlations with ADMA, as did the variables creatinine and total bilirubin with Met-SO. Hematocrit, hemoglobin, and INR as well as minimum and maximum oxygen saturations revealed significantly positive correlations with Met-SO/Met values, and oxygen uptake at the anaerobic threshold and maximum oxygen uptake displayed significantly negative correlations with Met-SO/Met values. Single significant correlations between isolated variables included positive correlation of albumin with ornithine and serine, of triglycerides with alpha aminoadipic acid, and of the oxygen uptake at the anaerobic threshold with threonine as well as negative correlations of alanine aminotransferase activity with taurine and of INR with serotonin. No further correlations were identified, especially none with CRP or NT-proBNP (Supplemental Table 1).

## Discussion

To the best of our knowledge, our study is the first clinical metabolomics study focusing on Fontan patients’ serum amino acid patterns. Its main finding is that, in comparison with controls, adult Fontan patients with a morphologically left dominant ventricle exhibit a distorted amino acid metabolome, hinting at altered (myocardial) cell energy metabolism and an elevated myocardial protein turnover as well as at oxidative stress and endothelial dysfunction, as found in biventricular patients with congestive heart failure.

### Altered cell energy metabolism and elevated myocardial protein turnover

Decreased serum concentrations of taurine, asparagine, and threonine and increased concentrations of glutamic acid and hydroxyproline in our Fontan patients indicate alterations in (myocardial) cell energy metabolism and elevated myocardium protein turnover, as found in biventricular patients with congestive heart failure. Taurine is abundant in myocardial tissue, with a major function in regulation of the respiratory chain: the taurine-deficient heart suffers impaired respiratory chain function and diminished long chain fatty acid uptake by mitochondria, as found in patients with congestive heart failure^10^. Decreased concentrations of taurine are described in patients with dilated cardiomyopathy and in golden retrievers with heart failure^10,11^, and many studies report a beneficial effect of taurine supplementation on myocardial function in patients with congestive heart failure^12^.

Glutamic acid and asparagine also take part in central pathways of aerobic cell respiration and thereby of energy production. Via anaplerotic reactions, both amino acids are substantially involved in the tricarboxylic acid cycle^13^. Against the background of a switch in heart failure of preferred myocardial energy substrate from fatty acids to glucose and ketone bodies^14^, our findings hint at altered myocardial energy metabolism, indicating subtle ventricular dysfunction. This idea is supported by glutamic acid’s involvement in the synthesis of metabolites, *e.g*., by serving as a precursor for the biosynthesis of amino acids such as proline and arginine^15,16^. Indeed, increased serum levels of glutamic acid indicate increased protein turnover, as typical in adults with congestive heart failure or coronary heart disease^17,18^. The same is true for threonine, a major component of proteins, *e.g*., of collagen and immunoglobulins: We found decreased serum threonine levels, and decreased plasma levels are described in patients with chronic heart failure^17^. The increase that we observed in hydroxyproline, an analyte used to track collagen degradation, also reflects elevated protein turnover: Elevated urine and serum hydroxyproline levels are reported after muscle damage or in the bedridden and elderly^19,20^.

### Oxidative stress and endothelial dysfunction

Protein turnover also is elevated in  the presence of hyperreactive oxygen species, *i.e*., under oxidative stress and during endothelial dysfunction, both of which are directly linked to heart failure: Nitrous oxide (NO) synthase (NOS) isoforms are expressed not only in endothelial cells but also in cardiomyocytes, and NO regulates cardiac function through vascular-dependent and -independent effects, with, in the healthy heart, a positive inotropic effect at low NO exposure and a negative one at higher exposure^21,22^. In heart failure, regulation of myocardial NO production and release breaks down, with excessive release, and peripheral and vascular endothelial NOS activity is lost, resulting in endothelial dysfunction with decreased NO bioavailability attributable to increased oxidative stress^23^. In the course of cardiac decompensation, NO likely influences several of the core features of cardiac failure, *e.g*., chamber dilation, defective b-adrenergic responsiveness, and calcium cycling^22^.

That serum concentrations of ADMA were elevated also suggests that our Fontan patients were under oxidative stress, with altered endothelial function, consistent with subtle ventricular dysfunction. ADMA, a methyl derivate of arginine, is involved in NO-signalling and in pro- and antioxidant and -inflammatory processes. It is the major endogenous inhibitor of nitric oxide synthase (NOS). By displacing arginine, the normal NOS substrate, ADMA enhances oxidative stress by influencing NO-reactive oxygen species balance and disturbs vasodilation^21,24–26^. ADMA values reportedly track enhanced cardiovascular risk with endothelial dysfunction^27,28^, positively correlating with age, mean arterial pressure, renovascular resistance, intimal media thickness, and peripheral arterial occlusive disease^5,29–33^, ADMA is additionally involved in further nitric oxide (NO)-dependent signalling processes, interfering with anti-thrombotic, anti-inflammatory, and anti-apoptotic actions^21^.

The increases in value for Met-SO and for the Met-SO/Met ratio as indicators of systemic oxidative stress also suggest that our patients are under oxidative stress and suffer from endothelial dysfunction. Reaction with oxygen species yields Met-SO, which activates endogenous antioxidant enzymes such as Met-SO reductase A and induces synthesis of glutathione, thereby counteracting oxidative stress and inflammation^34^. Increased Met-SO levels have been reported in vascular disease, cardiac ischemia, and in left ventricular diastolic dysfunction^35,36^. The negative correlation observed between Met-SO/Met values and oxygen uptake under exercise strikingly emphasizes the role of markers of oxidative stress as indicators for subtle ventricular dysfunction in our patients. Finally, our hypothesis that oxidative stress affects our patients is supported by the decreased level found of histidine, with its anti-oxidant and anti-inflammatory properties^37^, and by decreases in HDL-C^9,38–40^. A direct interplay of lipoprotein metabolism and markers for oxidative stress exists: lipoprotein disorders are associated with increased ADMA^41^.

ADMA also has been used to track heart failure in patients with CHD, and with regard to heart failure is even more sensitive than NT-proBNP. However, only 13% of the patients studied had univentricular heart disease; in addition, patient exercise capacity was lower than that in our patient group^33^. Thus we stress that our findings of altered amino acid serum levels might imply subtle rather than frank heart failure^42,43^, a hypothesis supported by the fact that we found no correlation of any of the metabolites with the traditional marker for heart failure, NT-proBNP, nor with the traditional marker for inflammation C-reactive protein, and by the fact that neither by traditional clinical-laboratory means nor by metabolomic (Fischer ratio) criteria did we find any evidence for important (Fontan-associated) liver disease in our patients. Given that many adult Fontan patients will develop ventricular dysfunction, to hypothesise subtle heart failure in our patient group might well be appropriate^1^. Still, with respect to the definition of heart failure, which is the “inability of the heart to meet resting and exercise demands at low filling pressures”^2^, we cannot exclude with certainty that the metabolic alterations delineated solely reflect Fontan-specific pathophysiology with its circulatory abnormalities, with paramount abnormal systemic or pulmonary endothelial function, and with a tendency towards the formation of veno-venous or aortopulmonary collaterals^44–48^.

To examine the amino acid profile of Fontan patients with frank ventricular, circulatory, or hepatic impairment and to correlate increases in serum ADMA and Met-SO content with direct measurement of endothelin or with collateral vessel flow via magnetic resonance imaging studies thus would be of interest^49,50^.

### Limitations

Because Fontan patients are few, the studied group size is small, possibly limiting the extent to which our findings can be generalized. Also limiting may be our choice of a targeted metabolomic approach and a commercial kit, as metabolites not assessed may be important. That we analyzed serum samples precludes direct comparison between our results and those of studies that used tissue samples, and we acknowledge that having assayed serum rather than vascular or myocardial tissue, we cannot rule out certain system-driven aspects of disease pathogenesis: That is, unknown confounders that affect metabolic profiles might be the true basis for the observed differences. Moreover, differences in body composition or lifestyle parameters might have influenced our results to an unknown degree. We strove to lessen the likelihood of such errors by following a strict inclusion and exclusion protocol, especially with regard to (known) comorbidities or medication.

## Conclusion

The striking alterations in amino acid profile that we found may link Fontan pathophysiology with altered cell energy metabolism, oxidative stress, and endothelial dysfunction as found in biventricular patients with congestive heart failure. Markers identified through mass spectrometry-based extended amino acid metabolism might thus complement traditional diagnostic tools such as imaging, exercise capacity testing, and traditional laboratory biomarker determinations, yielding a better understanding of Fontan pathophysiology, and they are promising candidates for the early detection of ventricular or circulatory dysfunction in Fontan patients.

## Methods

Like our recently published results on phospholipid and acylcarnitine metabolomic examinations, this work is a subwork of the main study protocol (Trial registration number: ClinicalTrials.gov Identifier NCT03886935)^9^.

### Patients

Between September 2016 and March 2017, we prospectively examined adult Fontan patients with a dominant left single ventricle and age- and sex-matched healthy biventricular controls at the Center of Pediatric Cardiology and Congenital Heart Disease, Heart and Diabetes Center North Rhine-Westphalia, Ruhr-University of Bochum, Germany^9^. All patients had undergone two-stage palliation with partial and total cavopulmonary anastomosis. None had had aortic reconstruction or aortopulmonary shunting. Table 4 shows inclusion and exclusion criteria. See the flow chart according to STROBE (Strengthening the Reporting of Observational Studies in Epidemiology [https://strobe-statement.org/index.php?id=strobe-home]) (Supplemental Fig. 1) for details on the flow of patients through the present study.

**Table 4:** Inclusion and exclusion criteria^9^.

Inclusion criteria	Exclusion criteria
Written informed consent	Medication affecting metabolic state
Age ≥18 years	Medication affecting hemodynamic state
8 hours fasting before blood sampling	Coronary artery disease
Dominant left ventricle (patients)	Failure of systemic ventricle
Biventricular healthy heart (controls)	Valvular stenosis
	Atrioventricular regurgitation > mild
	Aortic regurgitation > mild
	Recurrent effusions
	Protein-losing enteropathy
	Metabolic disease
	Malignancy
	Other cachectic disease, malnutrition
	Inflammatory disease
	Myeloproliferative disorder
	Pregnancy or lactation
	Multiple organ failure
	Mental or physical handicap precluding exercise
	$\dot{{\rm{V}}}$O_2_ AT < 20 ml/kg/min^7^

$\dot{{\rm{V}}}$O_2_AT, oxygen uptake at the anaerobic threshold.

Clinical and laboratory examinations were performed as described in detail elsewhere^9^. Age, sex, weight, medication, and cardiac risk factor assessment, with blood sampling for routine hematological and biochemical profiling, was conducted during an outpatient visit. Fasting patients underwent phlebotomy while recumbent. Echocardiography followed, and, after a defined snack rich in carbohydrates, exercise capacity was tested. We correlated metabolic results with routine laboratory parameters and exercise capacity parameters^51^. All patients underwent symptom-limited treadmill exercise capacity testing with expiratory gas analysis^52^. A 12-lead ECG was used to determine heart rate. Oxygen uptake at rest ($\dot{{\rm{V}}}$O_2_ at rest, ml/kg/min), at the anaerobic threshold ($\dot{{\rm{V}}}$O_2_AT, ml/kg/min), and maximum uptake of oxygen ($\dot{{\rm{V}}}$O_2_ max, ml/kg/min) were measured.

Blood studies required samples 0.5 ml greater than those for routine assessments to permit determinations of concentrations of amino acid-metabolism analytes. The blood sample was directly drawn into a tube containing a clotting activator and centrifuged within 20 min (15 °C, 10 min, 2500 × g) for separation of serum. Serum aliquots were immediately frozen and stored at -80 °C for further analyses. Frozen samples were transported on dry ice to the analysing laboratory. Analyses were performed in batches of 10 samples^9^.

### Sample preparation

Before analysis, all serum samples were processed as described, with samples thawed on ice, then centrifuged; the supernatant was subjected to further analyses^6,9^. The AbsoluteIDQ p180 kit assay (Biocrates Life Sciences AG, Innsbruck, Austria) permitted targeted, fully automated quantification of 188 analytes (42 amino acids or biogenic amines and their derivatives) based on phenylisothiocyanate derivatization in the presence of internal standards followed by liquid chromatography mass spectrometry using a TSQ-Vantage (Thermo Fisher Scientific, Waltham, MA) instrument with electrospray ionization.

### Statistical analysis

To exclude metabolites below the limit of detection (LOD), the raw data (µmol/L) were cleaned applying a modified 80% rule; for statistical analysis at least 80% valid values above LOD needed to be available per analyte in the samples for each group. This reduced the dataset to 143 analytes (29 amino acids or biogenic amines and their derivatives). Remaining values below LOD were imputed applying a logspline method with values between LOD and LOD/2. After log2 transformation of metabolomics data as well as of routine analytes and clinical data the dataset underwent multivariate (hierarchical cluster analysis) and univariate statistical analyses. Student’s t-testing with a Benjamini-Hochberg correction identified significant metabolite (and clinical routine-parameter) differences between patients and controls. P-values were calculated to identify significant changes between controls and Fontan patients, and were adjusted for multiple testing, or false discovery rate (FDR), according to Benjamini and Hochberg. To interpret the correlation, the significance of the correlation was calculated from r and from the degrees of freedom (a variable dependent on the sample number) (Supplemental Table 1). As a measure of linear correlation, r can have values between −1 and 1, where 1 indicates total positive linear correlation, 0 indicates no linear correlation, and −1 indicates total negative linear correlation. Correlations with FDR-adjusted p-values <0.05 and r > 0.5 (<−0.5) were considered statistically significant; in Supplemental Table 1, table cells displaying a statistically significant correlation are highlighted by a green background^9^.

### Ethical approval and informed consent

The study protocol was approved by the local ethics committees of the Medical University of Innsbruck, Austria (AN2015-0303 357/4.3), and of the Heart and Diabetes Center North-Rhine Westphalia, Ruhr University of Bochum, Germany (AZ 52/2016), the methods were carried out in accordance with the relevant guidelines and regulations, and the subjects gave written informed consent.

### Pathway analysis

Information about analytes and the pathways in which they are involved was based on https://www.metaboanalyst.ca/ and on the Kyoto Encyclopedia of Genes and Genomes (KEGG).

## Supplementary information

Supplemental Figure 1.

Supplemental  Table 1.

Supplemental Table  2.

## Data Availability

The datasets generated and analyzed during the current study are given as supplementary material (Supplemental Table 2).
