# Supplementary material for: Targeted metabolomic analysis of serum amino acids in the adult Fontan patient with a dominant left ventricle
Source: Sci Rep. 2020 Jun 2;10:8930. doi: 10.1038/s41598-020-65852-x (PMC7265548; doi:10.1038/s41598-020-65852-x)
Supplement: Supplementary file 1 — Supplemental Figure 1. [file 41598_2020_65852_MOESM1_ESM.pdf]

Supplementary information file – Supplementary figure 1

concerning the manuscript SREP-19-30992A with the title

"Targeted metabolomic analysis of serum amino acids in the adult Fontan patient with a dominant left ventricle"

authors

Miriam Michel, MD, Karl-Otto Dubowy, MD, Andreas Entenmann, MD, Daniela Karall, MD, Mark Gordian Adam, PhD, Manuela Zlamy, PhD, Irena Odri Komazec, PhD, Ralf Geiger, MD, Christian Niederwanger, MD, Christina Salvador, MD, Udo Müller, PhD, Kai Thorsten Laser, MD, and Sabine Scholl-Bürgi, MD

for consideration for publication in the journal "Scientific Reports"

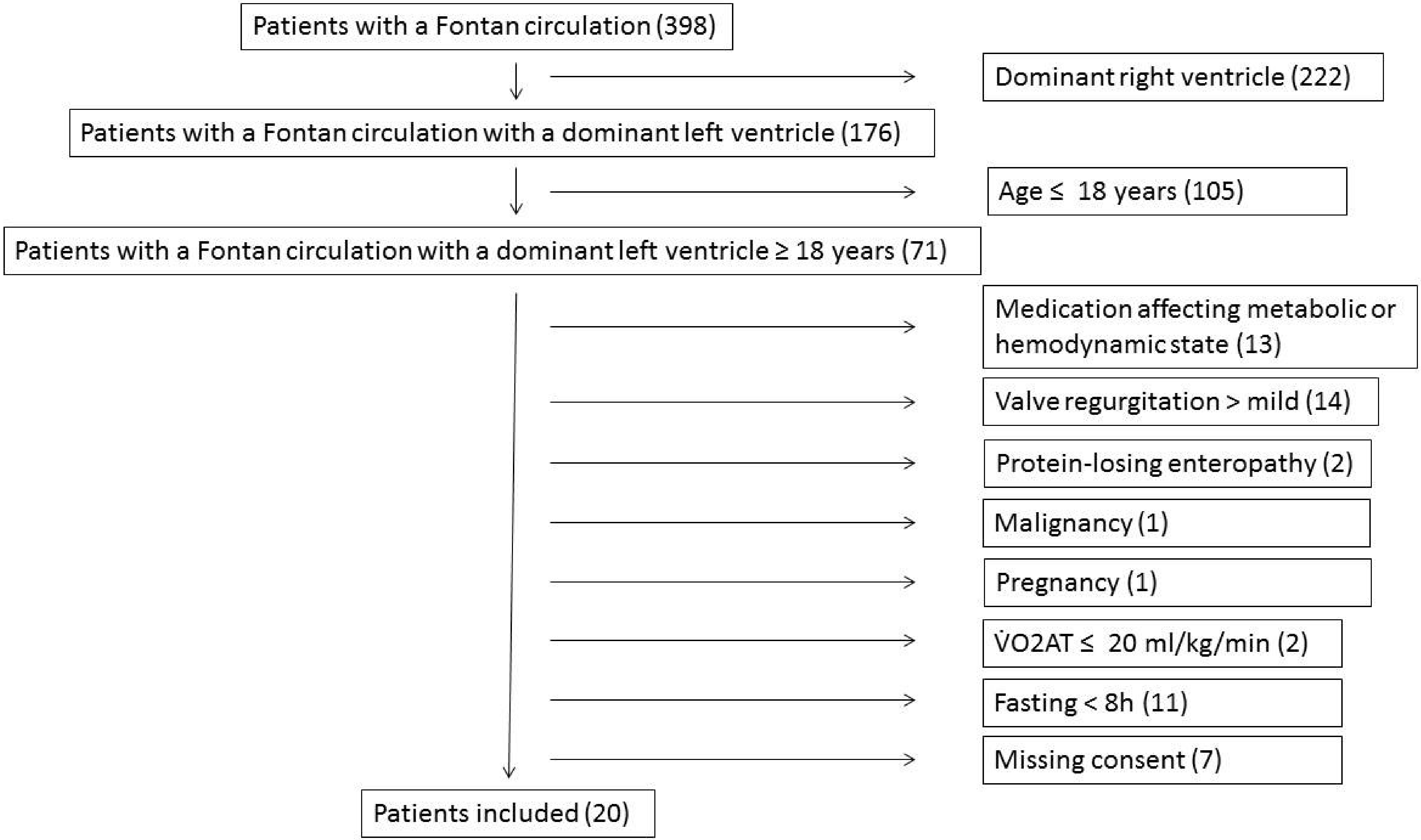
Patients with a Fontan circulation (398)
Dominant right ventricle (222)
Patients with a Fontan circulation with a dominant left ventricle (176)
Age ≤ 18 years (105)
Patients with a Fontan circulation with a dominant left ventricle ≥ 18 years (71)
Medication affecting metabolic or hemodynamic state (13)
Valve regurgitation > mild (14)
Protein-losing enteropathy (2)
Malignancy (1)
Pregnancy (1)
V̇O2AT ≤ 20 ml/kg/min (2)
Fasting < 8h (11)
Missing consent (7)
Patients included (20)
